# Transcriptomics of different tissues of blueberry and diversity analysis of rhizosphere fungi under cadmium stress

**DOI:** 10.1186/s12870-021-03125-z

**Published:** 2021-08-20

**Authors:** Shaopeng Chen, QianQian Zhuang, XiaoLei Chu, ZhiXin Ju, Tao Dong, Yuan Ma

**Affiliations:** 1 College of Agriculture, Jilin Agricultural Science and Technology University, Jilin, 132101 PR China; 2 Design Office, Jilin Greening Management Center, Jilin, 132011 PR China

**Keywords:** Blueberry, Cadmium, Transcriptomics, Microbial diversity, Heavy metal absorption

## Abstract

Blueberry (*Vaccinium* ssp.) is a perennial shrub belonging to the family Ericaceae, which is highly tolerant of acid soils and heavy metal pollution. In the present study, blueberry was subjected to cadmium (Cd) stress in simulated pot culture. The transcriptomics and rhizosphere fungal diversity of blueberry were analyzed, and the iron (Fe), manganese (Mn), copper (Cu), zinc (Zn) and cadmium (Cd) content of blueberry tissues, soil and DGT was determined. A correlation analysis was also performed. A total of 84 374 annotated genes were identified in the root, stem, leaf and fruit tissue of blueberry, of which 3370 were DEGs, and in stem tissue, of which 2521 were DEGs. The annotation data showed that these DEGs were mainly concentrated in a series of metabolic pathways related to signal transduction, defense and the plant–pathogen response. Blueberry transferred excess Cd from the root to the stem for storage, and the highest levels of Cd were found in stem tissue, consistent with the results of transcriptome analysis, while the lowest Cd concentration occurred in the fruit, Cd also inhibited the absorption of other metal elements by blueberry. A series of genes related to Cd regulation were screened by analyzing the correlation between heavy metal content and transcriptome results. The roots of blueberry rely on mycorrhiza to absorb nutrients from the soil. The presence of Cd has a significant effect on the microbial community composition of the blueberry rhizosphere. The fungal family Coniochaetaceae, which is extremely extremelytolerant, has gradually become the dominant population. The results of this study increase our understanding of the plant regulation mechanism for heavy metals, and suggest potential methods of soil remediation using blueberry.

## Introduction

Cadmium (Cd) is a toxic heavy metal that accumulates in soil as a result of air and water circulation, and acts on the human body through the food chain. The accumulation of Cd in the human body causes severe toxic reactions [1]. The pollution area and toxic effects of Cd have a serious impact worldwide [2]. At present, Cd pollution of agricultural soils in China is very extensive, and has caused major environmental problems [3]. Moreover, Cd and its compounds are readily soluble in water under acidic conditions. However, the dissolution rate of Cd in neutral to alkaline soil is very low [4]. Therefore, how to efficiently remove Cd in acid-contaminated soil is a very important topic [5, 6]. The use of hyperaccumulators for remediation of contaminated soil is highly efficient and protects the environment, and has therefore attracted increasing attention. Plant species such as *Rorippa globulosa*, *Solanum nigrum*, *Sedum alfredii*, *Viola baoshanensis* and *Pteris vittata* can enrich Cd and some other heavy metals in soil [7]. However, the use of plants for soil remediation is also associated with slow growth rates, resulting in economic losses.

Blueberry (*Vaccinium* ssp.) is a perennial shrub belonging to the family Ericaceae that thrives on acid soil. It was introduced and domesticated successfully in the early twentieth century. However, it is only within the last 20 years that, as the unique flavour and high nutritional value of this crop have led to growing demand for its production, its cultivation area has expanded rapidly worldwide, particularly in Europe, North America, South America and Asia, including China [8]. It is well documented in the literature that blueberry fruit is rich in anthocyanins, and contains among the highest antioxidant levels of any fruit [9]. Moderate intake can significantly reduce the risk of cardiovascular and cerebrovascular disease and type 2 diabetes mellitus, and provides some degree of neuroprotection [10]. However, a number of studies have highlighted a new potential application for this plant. Blueberry is a dominant tree species in acid soils polluted with heavy metal compounds [11, 12], to the extent that it is often used as an indicator species for monitoring local pollution with heavy metals [13, 14]. Kandziora-Ciupa et al. studied the response of *V. myrtillus* (bilberry) to heavy metals in three contaminated sites in Finland. Their results showed that *V. myrtillus* could adapt to the polluted soil [15]. A large number of plants of *V. angustifolium* and *V. myrtilloides* grow around the ore smelters in Sudbury, Canada. Both species can tolerate heavy-metal-contaminated soil and grow well on it. It is of paramount importance that the levels of heavy metal pollutants (Cu and Ni) in the fruits of these species do not exceed the internationally agreed safe values, so that they are safe for human consumption [16]. In the Majiang blueberry production area in China, Li et al*.* measured the heavy metal content of soil and fruit. Their results showed that although the heavy metal levels in soil exceeded the maximum safe value, the corresponding levels in fruit did not. Chen et al. studied blueberry plants that had been cultivated in Cd-contaminated soil, and found that the Cd content of the fruit was not significantly affected by soil pollution [17], which suggests a potential new role for this plant as a remediation species in acid soil contaminated with heavy metals.

The absorption and detoxification of heavy metals in plants are complex regulatory processes. Unnecessary heavy metals seriously impair morphological and structural development, inhibit physiological processes, cause oxidative stress reactions, and ultimately result in death of the plant [18–20]. Cotton (*Gossypium hirsutum*) can resist Cd toxicity by a combination of cell wall thickening, an increase in antioxidant activity and the formation of complexes with heavy metals under conditions of Cd stress, and there are also specific signal transduction pathways, such as the brassinosteroid and ethylene signalling pathways, in this species [21]. In response to Cd stress, ABC transporters, Zrt/IRT and transcription factors are significantly induced in the roots of *Festuca arundinacea*, mainly reflecting activity of the pathways related to glutathione metabolism, phenylpropanoid biosynthesis and nitrogen metabolism [22]. Compared with the high-Cd-accumulating type, there is a delay in transcriptional changes in response to Cd in the low-Cd-accumulating type. Cell wall biosynthesis and glutathione metabolism are involved in the Cd defense response of the high-Cd-accumulating type. Moreover, the expression of Cd transport genes has been found to differ between two types of Chinese cabbage [23]. In *Kandelia obovata* under Cd or Zn stress, three phenolic metabolism pathways are involved in the heavy metal tolerance mechanism, and the heavy metal content of the leaves is positively correlated with their total phenol content [24].

Heavy metals present in the soil not only affect the plants growing in it, but also have a significant impact on the microorganisms in the soil. In previous studies it has been reported that soil microorganisms are much more sensitive to heavy metal stress than are either soil animals or plants growing in the same soil [25]. Excessive amounts of heavy metals in the soil also lead to changes in the biomass and diversity of soil microorganisms [26]. A combination of plant root exudates and the microorganisms associated with the roots can enhance the plant’s tolerance of the toxic effects of heavy metals [25]. It has been found that soil microorganisms can reduce the mobility and bioavailability of metals through the processes of biosorption and precipitation [27]. The degree of severity of heavy metal pollution also affects the composition of the microbial community. Under conditions of severe pollution, the dominant microbial population in rice roots is positively correlated with Cd concentration [28]. Blueberry has a small fibrous undeveloped root system, which forms mycorrhizal associations with mycorrhizal fungi that also interact with symbiotic bacteria and provide the necessary nutrients for the plant [29]. The mycorrhizae of Blueberry can help the plant to resist the adverse effects of environmental factors such as drought and low temperature [30, 31].

To date there have been only a few reports on the physiological effects of heavy metals on blueberry cultivation. Blueberry can reduce the toxic effects of heavy metals by regulating its own antioxidant stress response. The toxic effects are related to the type of heavy metal and the duration of exposure. It has also been found that the levels of nitrogen, phosphorus and potassium in blueberry are positively correlated with the plant’s heavy metal content, and that the glutathione (GSH) content of blueberry decreases under excessive stress [15, 32]. However, the mechanism of heavy metal tolerance, the relationship with tissue redistribution, and even the changes in mycorrhizal fungi in blueberry are unclear. Therefore, in order to explore the response of blueberry to heavy metals, as well as the changes in mycorrhizal fungi under Cd stress, there is a need to understand the internal regulation mechanism for heavy metals in this plant, and to explore the potential use of blueberry for remediation of acid soil that is contaminated with heavy metals.

## Test materials and methods

### Test materials and pretreatment

*Vaccinium corymbosum* ‘Bluegold’, a late-maturing variety of blueberry grown on the horticultural farm of Jilin Agricultural Science and Technology University, was used as the experimental material for this study. In May 2017, 2-year-old blueberry seedlings were planted in a non-woven pot (50.0 cm × 50.0 cm × 50.0 cm). The planting medium was peat soil, and the soil pH was adjusted to 5.0 with sulfur. Blueberry plants were normally maintained in the pot for two growth cycles to ensure that they developed up to the high-yield stage. On 1 June 2020, blueberry plants with the same rate of growth, normal pollination and fruit setting were selected for Cd stress testing. In order to avoid the interference of Cl^–^ ions with plant growth, the treatment group was treated with an aqueous solution of CdSO_4_ to give a soil Cd^2+^ concentration of 400 mg/kg (calculated on the basis of the weight of pure metal), and the control group (CK) was potted without CdSO_4_. Each treatment was repeated three times. After the potted plants had been treated, they were all maintained in full sunlight in a greenhouse to prevent leaching by rainwater. Management consisted of irrigation of each basin with 3.0 L of deionized water every 2 days, and the leachate under the basin was collected for reinjection. Blueberry fruits were harvested on 25 August 2020, and roots, stems, leaves and soil samples were collected on 30 September 2020. All tissue samples were washed with deionized water three times and dried in the shade. They were then frozen in liquid nitrogen and stored in aseptic bags at –80℃ in a freezer. After the blueberry plant roots had been dug up and excess soil removed from them, they were shaken in sterile self-sealing bags so that the remaining soil attached to the roots was separated out. The roots were then frozen in liquid nitrogen and stored in a freezer at –80℃.

### Construction of transcriptome database

#### RNA extraction

Total RNA was extracted from the roots, stems, leaves and fruits using the Total RNA Purification Kit, TRK1001 (LC Science, Houston, TX), and was then subjected to agarose gel electrophoresis prior to testing. The quality and concentration of samples was checked using the Agilent 2100 Bioanalyzer (Agilent, USA). The O.D. A260/280 was above 1.70–2.10, and the concentration was between 0.03–0.23 µg/µL.

#### Database construction, sequencing and annotation

Total RNA was extracted from roots, stems, leaves and fruits of the treatment group and the control group for transcriptome sequencing. The sequencing included three biological replicates, giving a total of 24 groups of samples. The sequencing work was undertaken by Hangzhou Lianchuan Bio Technologies Co., Ltd., and the NovaSeq 6000 System (Illumina, USA) was used for sequencing. In order to maximize the quality of data, the original data were filtered. The content of filtering includes: removing the splice sequence from the sequencing reads; The sequencing reads were scanned by window method, and the default scanning window was 6 bp, When the average quality value in the window is less than 20, the part of the read from the beginning of the window to the end of 3 ‘is cut off; Poly-A / T was removed; The truncated sequences less than 100 bp in length were removed; Remove the truncated sequence with more than 5% N content The sequence quality, including effective sequencing quantity, Q20, q30 and GC content, was estimated using FastQC (http://www.bioinformatics.babraham.ac.uk/projects/fastqc/). De novo assembly of the transcriptome was performed with Trinity 2.4.0. All the newly assembled gene sequences (UniGene) were compared with the NR database (http://www.ncbi.nlm.nih.gov/), GO (http://www.geneontology.org), SwissProt (http://www.expasy.ch/sprot/), KEGG (http://www.genome.jp/kegg/), Pfam(http://pfam.xfam.org/) and EggNOG (http://eggnogdb.embl.de/) Compare and annotate the database.

#### Differentially expressed Unigene analysis

Salmon was used to perform expression level for Unigenes by calculating TPM. The differentially expressed Unigenes were selected with log2 (fold change) > 1 or log2 (fold change) < -1 and with statistical significance (*p* value < 0.05) by R package edgeR [33–35].

#### Fluorescence-based quantitative PCR

The transcription levels of differentially expressed genes (DEGs) in roots, stems, leaves and fruits were determined by quantitative real-time PCR (qRT PCR), which was performed using SYBR Premix Ex Taq (Tli RNase H plus, Takara). cDNA (20 times diluted) was used as the template for qRT PCR. The operation process and reaction conditions were as described in the manufacturer’s instructions, and the qRT PCR was repeated three times. The relative gene expression was calculated using the comparative CT (^−ΔΔ^CT) method. The gene-specific primers for qRT PCR were designed using Primer Express Software v3.0, and the internal reference gene was blueberry glyceraldehyde-3-phosphate dehydrogenase (GAPDH, NCBI accession number: AY123769.1). The qRT PCR specific primers and internal reference primers are listed in Table 1.

**Table 1:** Specific primers and internal reference primers

Primer name	Sequence (5ʹ-3ʹ)	Description
DN28053_c0_g3-F	TGGGCTCTATGTAAATCTCCGTAT	Root DN28053 c0 g3 qRT-PCR Primer
DN28053_c0_g3-R	CAACTTGCTTATTCCACCATACTCAT	Root DN28053 c0 g3 qRT-PCR Primer
DN43627_c1_g2-F	GGGATTTGTTTAGGGTAGTTGAAGG	Root DN43627 c1 g2 qRT-PCR Primer
DN43627_c1_g2-R	TGAGACCAATGTCCCAACCACT	Root DN43627 c1 g2 qRT-PCR Primer
DN32241_c0_g1-F	CAAAGCCTCCTATGGGTCTGA	Root DN32241 c0 g1 qRT-PCR Primer
DN32241_c0_g1-R	TATGACCGTGGCAAGGTCTG	Root DN32241 c0 g1 qRT-PCR Primer
DN21228_c0_g1-F	TTTCGGACATCCAAAGACAGC	Root DN21228 c0 g1 qRT-PCR Primer
DN21228_c0_g1-R	GAAGAGTGCCTGTATGAGGGTTG	Root DN21228 c0 g1 qRT-PCR Primer
DN28398_c1_g2-F	ATGAATGGAGGGCTTGGAGAA	Root DN28398 c1 g2 qRT-PCR Primer
DN28398_c1_g2-R	CAGCCCAAGTCAGCAATGTTT	Root DN28398 c1 g2 qRT-PCR Primer
DN29181_c0_g2-F	CGGGTCAGTTTACGAGCAGAA	Stem DN29181 c0 g2 qRT-PCR Primer
DN29181_c0_g2-R	GCACATACGCCATCTTCTTCG	Stem DN29181 c0 g2 qRT-PCR Primer
DN35069_c1_g1-F	GTGGACCCAAAGCAGCACAAG	Stem DN35069 c1 g1 qRT-PCR Primer
DN35069_c1_g1-R	GCCAGGTTGGTAATCTGAGGG	Stem DN35069 c1 g1 qRT-PCR Primer
DN39782_c0_g2-F	GGTCGCTTTCCTCCACTCA	Stem DN39782 c0 g2 qRT-PCR Primer
DN39782_c0_g2-R	ACGCCAAACTCAAAGCCAGA	Stem DN39782 c0 g2 qRT-PCR Primer
DN35079_c1_g3-F	CCCCACATTAGCGGTGTTCAA	Stem DN35079 c1 g3 qRT-PCR Primer
DN35079_c1_g3-R	CTTCACGTCCAAAACACTTAGCTTC	Stem DN35079 c1 g3 qRT-PCR Primer
DN36954_c0_g2-F	GTGCCCTAACTTTGGGATGACT	Stem DN36954 c0 g2 qRT-PCR Primer
DN36954_c0_g2-R	CATCATTCCTTTCCAAGCACC	Stem DN36954 c0 g2 qRT-PCR Primer
DN41993_c0_g1-F	CTACGACGCACTTGCCTCTGT	Leaf DN41993 c0 g1 qRT-PCR Primer
DN41993_c0_g1-R	GGGCAAACTGAAGAGGCACAT	Leaf DN41993 c0 g1 qRT-PCR Primer
DN30310_c0_g3-F	TAGTTTCCGTTTGGGTATGCG	Leaf DN30310 c0 g3 qRT-PCR Primer
DN30310_c0_g3-R	TTGTGCTCAGCCTGGAATACG	Leaf DN30310 c0 g3 qRT-PCR Primer
DN44211_c1_g3-F	ATGAAGTATGGCGGCTGGAAA	Leaf DN44211 c1 g3 qRT-PCR Primer
DN44211_c1_g3-R	TGTTCTACGGTAAACTCCCACATC	Leaf DN44211 c1 g3 qRT-PCR Primer
DN26309_c0_g1-F	CGTCGGGAATGATTGGAGTTT	Leaf DN26309 c0 g1 qRT-PCR Primer
DN26309_c0_g1-R	TCTTCTTTCAGCCCGTAATCG	Leaf DN26309 c0 g1 qRT-PCR Primer
DN42843_c0_g4-F	TTTGAGTGATAAGATGCCGTTCC	Leaf DN42843 c0 g4 qRT-PCR Primer
DN42843_c0_g4-R	TCCAGAGTGGTTCCGAGTAGGT	Leaf DN42843 c0 g4 qRT-PCR Primer
DN29906_c1_g1-F	GCCGCTGCTGGTTCCATTTA	Fruit DN29906 c1 g1 qRT-PCR Primer
DN29906_c1_g1-R	GTTGCCAAATGCCCAATACCC	Fruit DN29906 c1 g1 qRT-PCR Primer
DN30439_c2_g2-F	GTGACCCTCCAAACCTCCATT	Fruit DN30439 c2 g2 qRT-PCR Primer
DN30439_c2_g2-R	GGCAGTTCTTGAGCCCTTGAT	Fruit DN30439 c2 g2 qRT-PCR Primer
DN44638_c1_g4-F	CGTCTCCTCAACTGCCCTCTT	Fruit DN44638 c1 g4 qRT-PCR Primer
DN44638_c1_g4-R	CTCCAGCAATGGCACATCTTT	Fruit DN44638 c1 g4 qRT-PCR Primer
DN21435_c0_g1-F	CTTGACACCGAGGCTGCTTAT	Fruit DN21435 c0 g1 qRT-PCR Primer
DN21435_c0_g1-R	TTGATGACCTGAAACGCCACA	Fruit DN21435 c0 g1 qRT-PCR Primer
DN43035_c0_g1-F	TTTGAATAAGGGTGATTTGAGTGTC	Fruit DN43035 c0 g1 qRT-PCR Primer
DN43035_c0_g1-R	TTCAACAACGAAGCCAATACAA	Fruit DN43035 c0 g1 qRT-PCR Primer
GAPDH-F	CATCCACTCTATCACCGCAACAC	Reference gene qRT-PCR Primer
GAPDH-R	GCAGGCAACACCTTACCAACAG	Reference gene qRT-PCR Primer

### Determination of heavy metal content

The Cd, Mn, Cu, Fe and Zn content of roots, stems, leaves and fruits was determined by inductively coupled plasma mass spectrometry (ICP-MS). The specific operation mode was as described in *GB 5009.268–2016 National Food Safety Standard: Determination of Multi Elements in Food*. The microwave digestion method was used to measure the Cd, Mn, Cu, Fe and Zn content in soil according to the specific operation mode described in *HJ 832–2017 Microwave Digestion Method for Total Metal Elements in Soil and Sediment*.

### DGT (diffusive gradients in thin films) technology

DGT technology was used to simulate the absorption of heavy metals in soil by plant roots. A 200 g sample of soil taken from the middle of the basin was dried at 85℃ to constant weight, and was then passed through a 2 mm sieve to remove large particles. Deionized water was added to achieve 70% of maximum field capacity, and was mixed well with the soil. The soil was sealed with plastic film and placed in an incubator at 25℃ for 48 h to balance the soil. The balanced soil was then evenly smeared into the inner cavity of the DGT ring to ensure the same amount of soil was loaded into all of the DGT cavities. After loading, the soil was placed in an incubator at 25℃ for enrichment for 24 h before the recovery device. After the soil had been cleaned with deionized water the fixed membrane was removed, the front of the membrane was rolled into a 2.0 mL centrifuge tube containing 1.8 mL of 1.0 M HNO_3_) for 24 h [36], and the heavy metal content was determined at the end of the extraction. Colorimetric analysis was used to determine the ferrous iron (Fe^2+^) content [37], and ICP-MS was used to determine the levels of the other heavy metals.

### Diversity of fungi in the rhizosphere

Fungal DNA was extracted from the rhizosphere of blueberry using the Total TopTaq DNA Polymerase Kit (TransGen Biotech Co., Ltd, China), and total fungal DNA was then subjected to agarose gel electrophoresis prior to testing. The quality and concentration of DNA samples were checked using the NanoDrop 2000 spectrophotometer. The concentration was higher than 20 ng/ L, the total amount was more than 500 ng, and OD260/280 was in the range of 1.8 ~ 2.0. DNA samples were stored in a freezer at –80℃. After the total DNA of rhizosphere fungi had been extracted, the primers were designed according to the conserved region, and the sequencing connector was added at the end of the primers. After PCR amplification, the single band was cut and recovered for library quality inspection. The library was then sequenced using the Illumina MiSeq platform and a 2 × 250 bp paired-end sequencing strategy. For operational taxonomic unit (OTU) analysis and species annotation, UCLUST in QIIME2 (version 1.8.0) software was used [38, 39]. After filtering, de-noising, splicing and removal of chimeras, OTUs were clustered at a similarity threshold of 97% and compared with the fungal taxonomy database (UNITE, http://unite.ut.ee/index.php). Finally, taxonomic annotation at the level of phylum, class, order, family, genus and species was performed. The species annotation, alpha diversity, Simpson and Shannon indices were analyzed using krona, Mothur v.1.30 (http://www.mothur.org/).

### Data analysis and mapping

SPSS 22.0 was used for data analysis, and Excel and R were used to plot the data.

## Results

### Transcriptome database analysis

#### Gene assembly data

A total of 24 blueberry cDNA libraries were sequenced using the NovaSeq 6000 platform. After filtering and assembling the original data (Table 2) a total of 84 374 genes were obtained, with a GC content of 42.02%. The shortest gene sequence was 201 bp, and the longest sequence was 15 879 bp, with an average length of 491 bp and N50 length of 1473 bp. This indicates that the sequencing quality meets the requirements and can be used for subsequent analysis.

**Table 2:** Gene assembly data

Index	All	GC%	Min Length	Median Length	Max Length	Total Assembled Bases	N50
Transcript	271,500	42.08	201	632	15,879	257,120,425	1465
Gene	84,374	42.02	201	491	15,879	72,538,271	1473

#### Gene annotation data

A total of 84 374 genes were annotated, and the results are shown in Table 3.

**Table 3:** Gene annotation data

Database	Number	Ratio (%)
All	84,374	100
GO	31,729	37.61
KEGG	25,247	29.92
Pfam	28,741	34.06
SwissProt	25,692	30.45
EggNOG	36,070	42.75
NR	37,714	44.7

#### Differential expression of genes

**Fig. 1:**
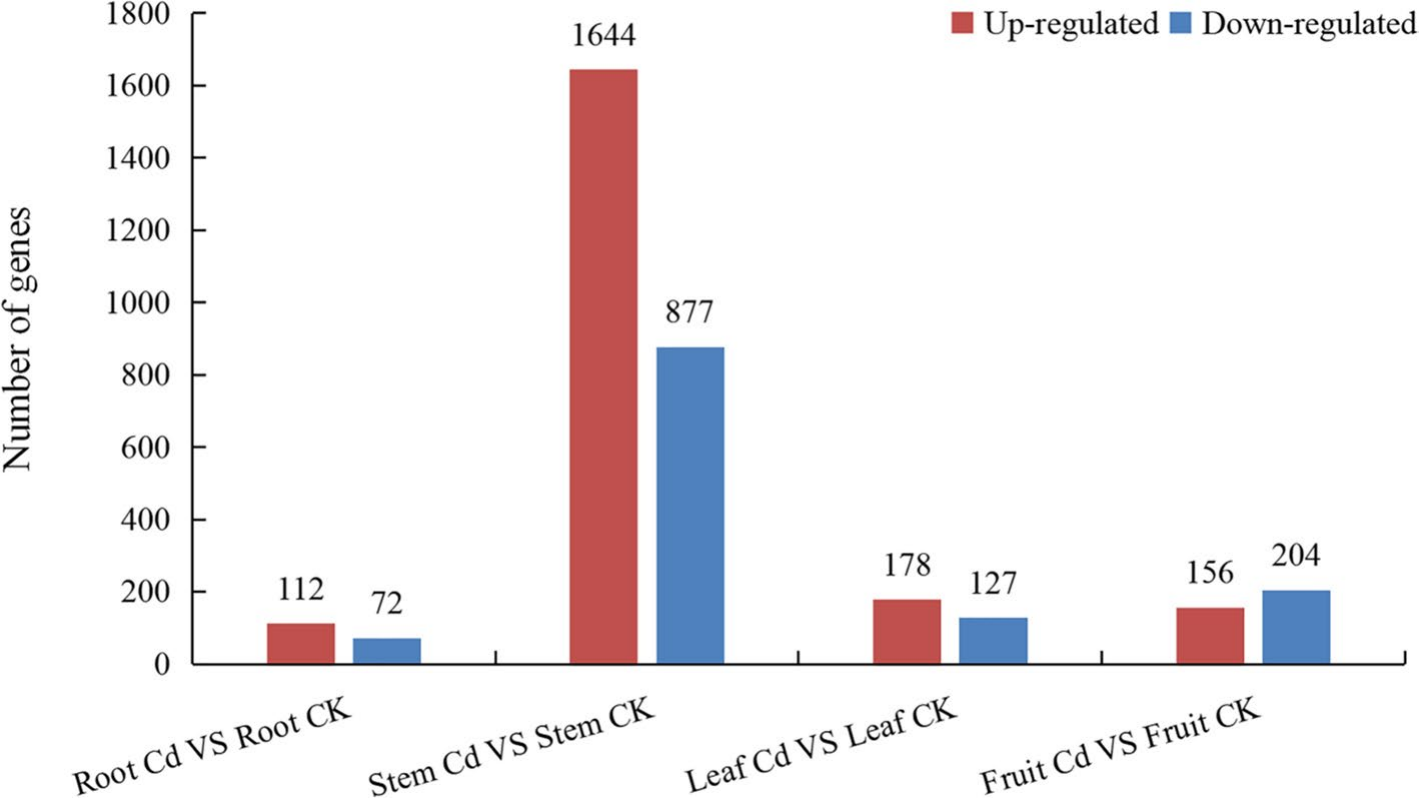
Differentially expressed genes in different tissues of blueberry

The DEGs in different tissues of blueberry that had been treated with Cd are shown in Fig. 1. There were 3370 DEGs in root, stem, leaf and fruit tissue (*P* < 0.05), of which 2521 DEGs (1644 up-regulated genes and 877 down-regulated genes) were present in stem tissue (representing 74.81% of the total). There were 360 (10.68%) DEGs in fruits, 305 (9.05%) DEGs in leaves, and 184 (5.46%) DEGs in roots.

#### Verification of differentially expressed genes by qRT PCR

**Fig. 2:**
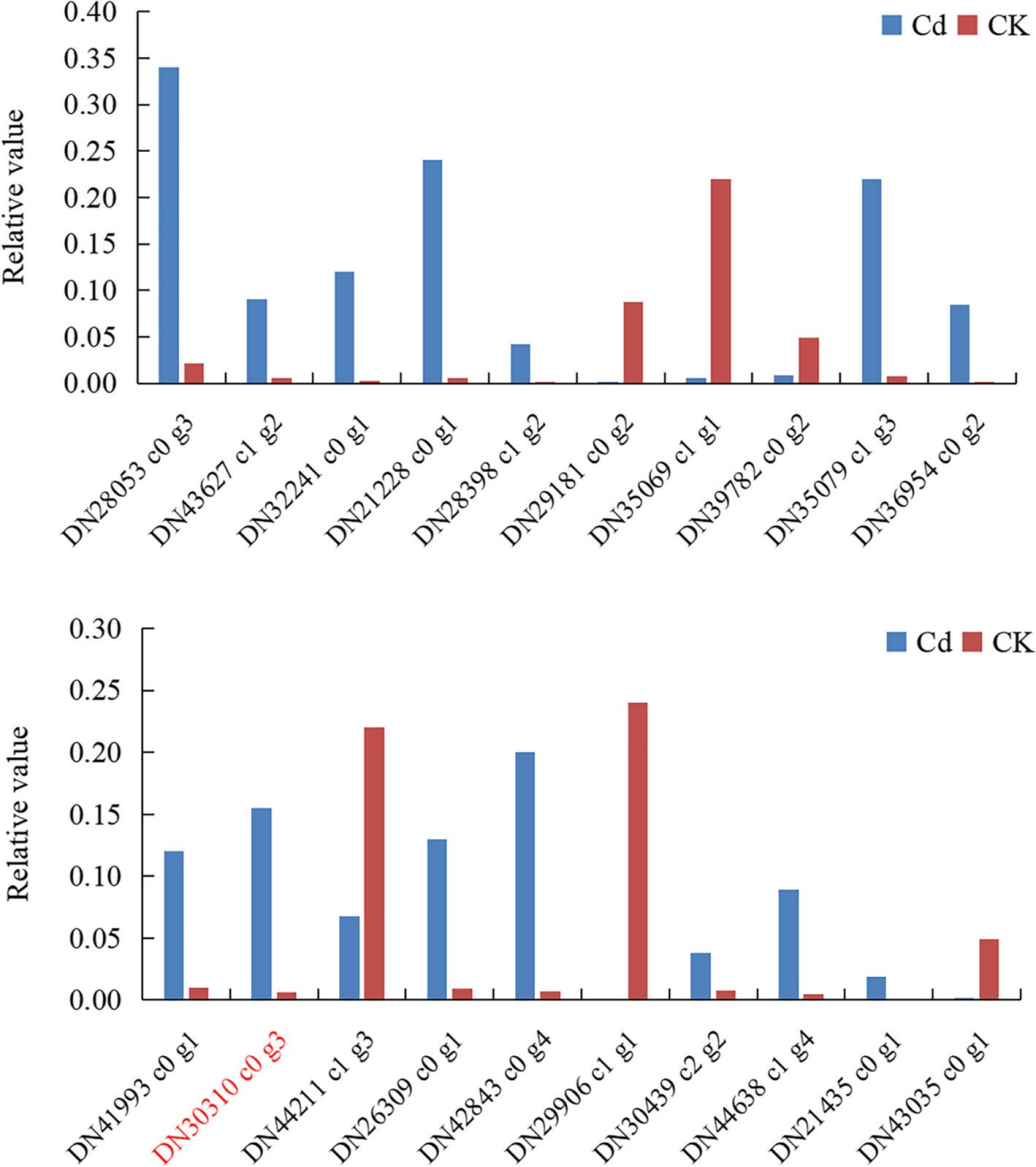
The 20 differential genes that were verified by qRT-PCR

In order to verify the reliability and reproducibility of RNA-Seq, 20 genes were randomly selected from blueberry root, stem, leaf and fruit tissue for qRT PCR detection, and the expression of these genes in different tissues under Cd stress was analyzed (Fig. 2). They included functional genes, transporters, protective enzymes and transcription factors. The expression of 20 differential genes was consistent with that of RNA-Seq, with the exception of dn30310 c0g3 (shown in red), which indicated that the sequencing results were reliable.

#### Gene ontology (GO) enrichment analysis

**Fig. 3:**
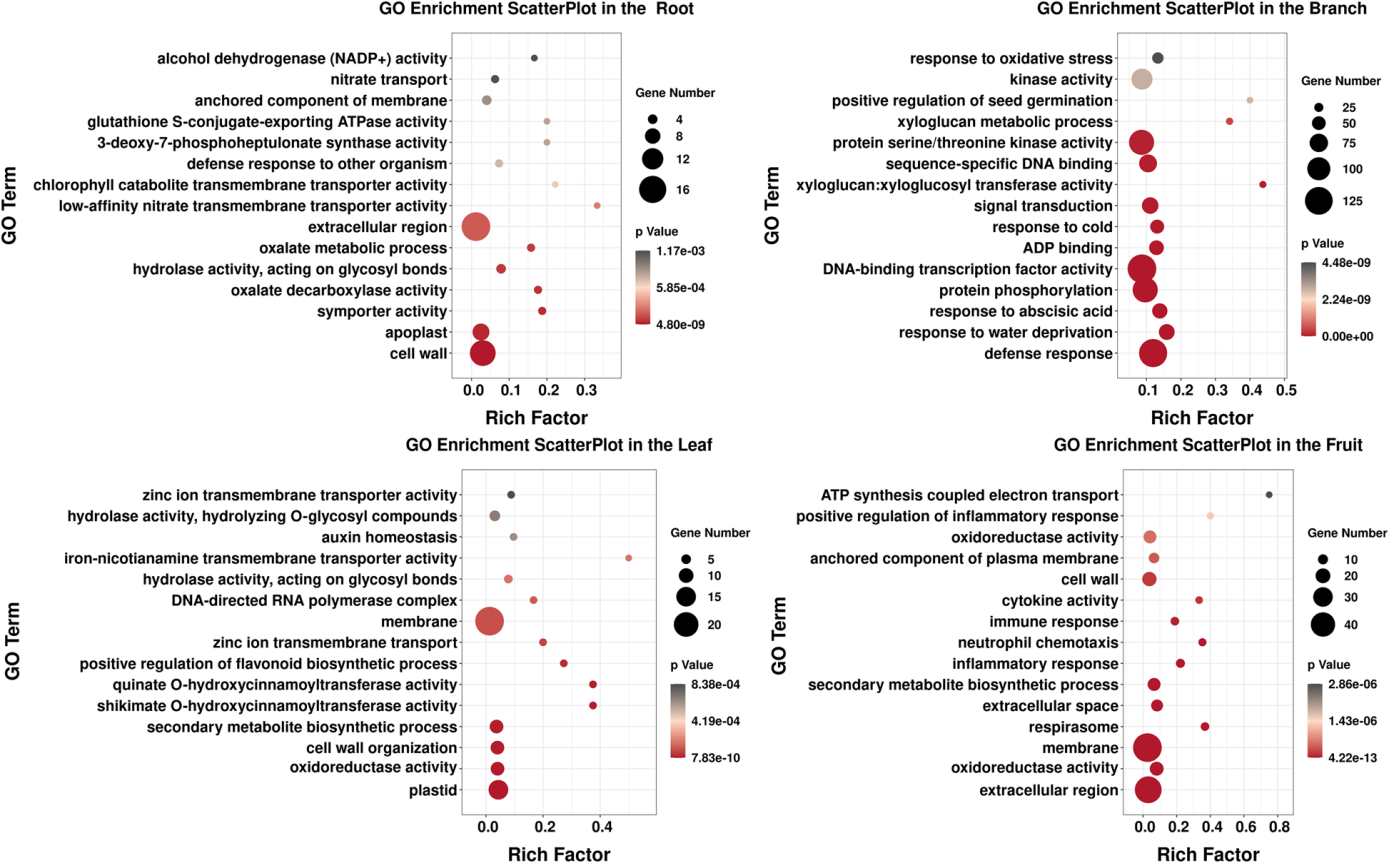
Enrichment analysis of GO genes in different tissues of blueberry

Under Cd^2+^ stress, the GO enrichment results for DEGs varied in different tissues of blueberry (Fig. 3), and only the top 15 *P*-values are shown in Fig. 3. A total of 96 significant GO-rich sub-items were obtained in root tissue, which were mainly concentrated in the cell wall, extracellular region and ectoplasm. In addition, the enrichment factors of cotransporter, oxalate metabolism and oxalate decarboxylase process were higher. A total of 226 significant GO-rich sub-items were obtained in stem tissue, which were mainly concentrated in the plasma membrane, and were also enriched in the defense reaction, abscisic acid reaction, protein phosphorylation, xyloglucosan and signal transduction pathways. A total of 107 significant GO-rich sub-items were obtained in leaf tissue, which were mainly concentrated in the cell membrane, extracellular region and plastids. The biosynthesis of secondary metabolites, cell wall tissue and oxidoreductase activity also played an important role. A total of 355 significant GO-rich sub-items were obtained in fruit tissue, which were mainly concentrated in the cell membrane and extracellular region. There were also many differences in the cell wall and in oxidoreductase activity.

#### KEGG enrichment analysis

**Fig. 4:**
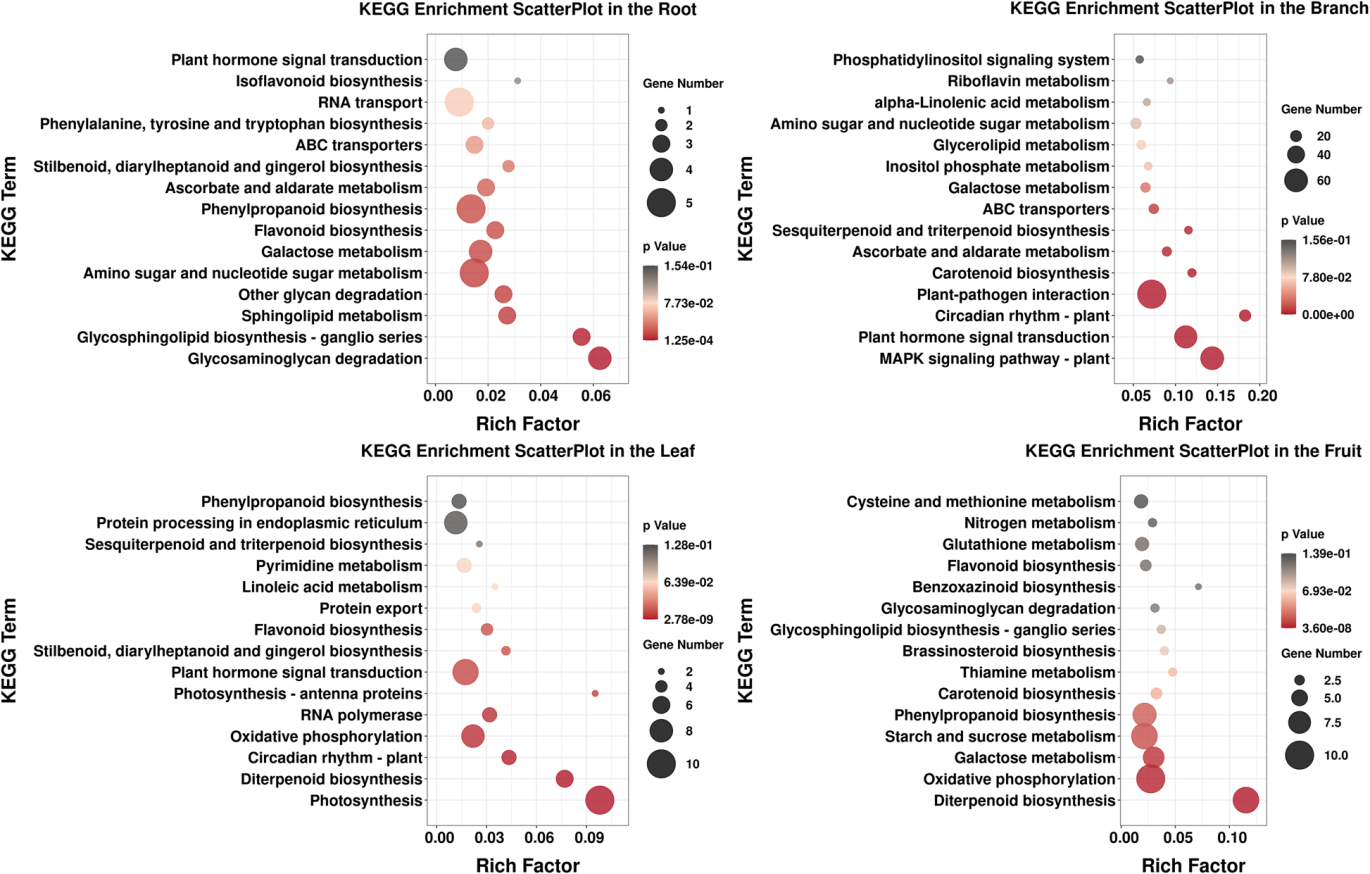
Enrichment analysis of KEGG in different tissues of blueberry

Under Cd stress, the KEGG enrichment results varied for different tissues of blueberry (Fig. 4). In terms of the number of differential genes, enrichment factor and *P*-value, the differential expression of the KEGG pathway in blueberry roots mainly involves sphingolipid metabolism, glycosphingolipid biosynthesis and glycosaminoglycan degradation and other processes related to the cell membrane and the cell wall, and the degradation and metabolism of lactose, amino sugars, nucleotide sugars and other glycans. In addition, it is associated with phenylpropanoid biosynthesis, flavonoid biosynthesis and RNA transport. There were also differences in metabolic processes such as ABC transport and plant hormone signal transduction. The difference in the KEGG pathway in blueberry stems was mainly concentrated in the plant–pathogen interaction pathway, and the difference in the plant hormone signal transduction and MAPK signal transduction pathways also reached a significant level. In blueberry leaves, the KEGG pathway was mainly expressed in photosynthesis, followed by plant hormone signal transduction and oxidative phosphorylation. The differentially expressed KEGG pathways in blueberry fruits were mainly involved in the biosynthesis of melamine, piperidine and pyridine alkaloids, thiometabolism, flavone and flavonol biosynthesis, ABC transport and purine metabolism.

### Heavy metal content in different tissues

**Fig. 5:**
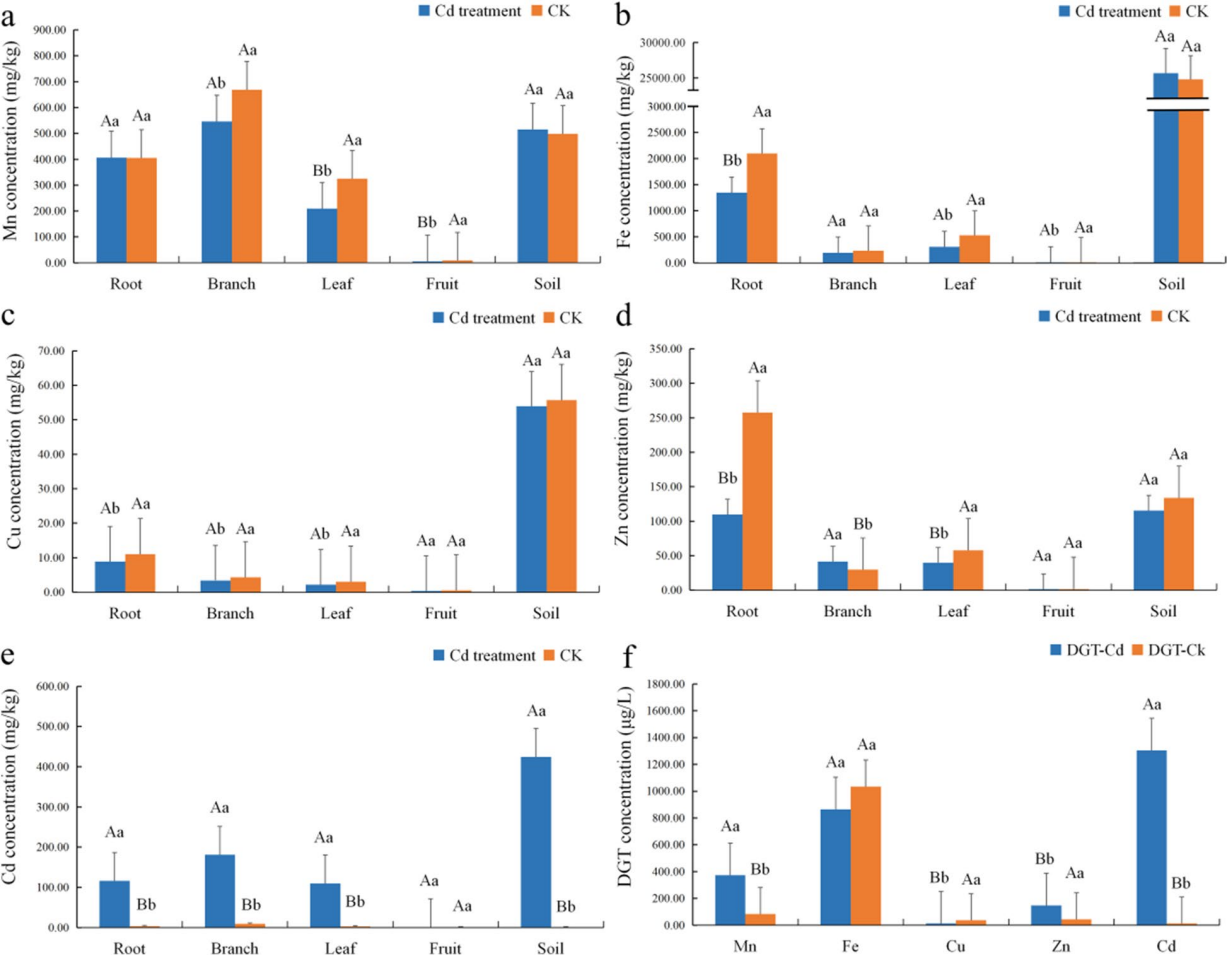
Heavy metal content of different tissues and DGT concentration in blueberry

Under Cd stress, the absorption of heavy metals by blueberry was significantly affected. It can be seen from Fig. 5A that Mn accumulated mainly in the root and stem of blueberry. Compared with the control group, the Mn content of root tissue in the treatment group was unaffected by Cd stress, and the Mn content of stem, leaf and fruit tissues decreased. The Mn content of leaf and fruit tissue in the treatment group was significantly different from that in the control group. The concentration of Mn was highest in the stem, where it exceeded Mn levels in the soil. Fe was the most abundant mineral element in the soil (Fig. 5b), and was mainly accumulated in the roots of blueberry. Under Cd stress, the Fe content of roots, stems, leaves and fruits decreased, and the Fe content of root tissue was significantly different compared with the control. The Cu content of blueberry tissues was much lower than the soil Cu content (Fig. 5C). Cd stress had no significant effect on the Cu content of blueberry tissues. The Cu content of root, stem and leaf tissue in the treatment group was significantly different from that in the control, but there was no significant difference in the Cu content of fruit tissue. The root system of blueberry has a significant enrichment effect with regard to Zn (Fig. 5d), and high concentrations of Zn can accumulate in the root tissue. Under Cd stress, the Zn content of root and leaf tissue decreased significantly, whereas the Zn content of stem tissue increased. There was no significant difference in the Zn content of fruit tissue between the treatment and control groups.

Cd is a non-essential element in plants, and under conditions of Cd stress the extent to which it accumulated in root, stem, leaf and fruit tissue of blueberry varied (Fig. 5e). It mainly accumulated in the stems and roots, and the highest levels were found in the stems, followed by the leaves. Compared with the control, there was a highly significant difference in the Cd content of roots, stems and leaves in the treatment group, but a much smaller difference in the Cd content of fruits. The total Cd content of roots, stems, leaves and fruits did not exceed soil levels.

DGT is a passive soil sampling technology that simulates the process of passive absorption of mineral elements by plant roots. It can be seen from Fig. 5F that Cd showed the highest rate of absorption by DGT, followed by Fe. Under Cd stress, there is a very significant increase in absorption of Mn and Zn compared with the control. Cu showed the lowest rate of passive absorption, and Cd impaired the absorption of Cu by DGT.

### Correlation analysis of heavy metal content and DEGs

The results of the correlation analysis of heavy metal content and DEGs in different tissues are presented in Table 4 (only the top 10 genes in each tissue are shown), and *R* ≥ 0.8 and *P* ≤ 0.05 were selected as the screening conditions. Of the 184 DEGs in blueberry root tissue, 37 DEGs were screened, including Cd30 (16.30%), Cu4 (2.17%), Zn2 (1.09%) and Fe1 (0.54%). Of these 37 DEGs, 16 DEGs were positively correlated and 21 DEGs were negatively correlated, and these genes included TRX2 and transcription factor myb26, among others. Of the 2521 DEGs in blueberry stem tissue, 1280 DEGs were screened, including Cd1230 (48.96%), Zn13 (0.52%), Fe5 (0.23%), Mn5 (0.23%) and Cu2 (0.09%). Of these 1255 DEGs, 762 DEGs were positively correlated and 493 DEGs were negatively correlated. including calmodulin-dependent protein kinase and L-ascorbate oxidase. Of the 305 DEGs in blueberry leaf tissue, 181 DEGs were screened, including Cu109 (35.74%), Cd48 (15.74%) and Mn24 (7.87%). Of these 181 DEGs, 100 DEGs were positively correlated and 81 DEGs were negatively correlated, including flavonoid 3-O-glucosyltransferase and peroxidase. Of the 360 DEGs in blueberry fruit tissue, 120 DEGs were screened, including Cu49 (13.61%), mn35 (9.72%), Zn17 (4.72%), Fe12 (3.33%) and Cd7 (1.94%). Of these 120 DEGs, 82 DEGs were positively correlated and 38 DEGs were negatively correlated, including rhamnosyltransferase and heavy-metal-associated isolated plant protein.

**Table 4:** Correlation analysis of heavy metal content and DEGs in different tissues

Tissue	Gene_ID	Related heavy metals	Length (bp)	Annotation	Species
Root	DN28710_c0_g3 +	Cd	700	Thioredoxin-like	*Actinidia chinensis*
	DN29606_c1_g1 +	Cd	902	LOW-QUALITY PROTEIN: probable LRR receptor-like serine/threonine-protein kinase At1g56130	*Herrania umbratica*
	DN31138_c0_g2-	Zn	2017	Butyrate–CoA ligase AAE11, peroxisomal-like	*Actinidia chinensis*
	DN24062_c1_g1-	Cd	1271	PREDICTED: receptor-like protein 12 isoform X2	*Juglans regia*
	DN23316_c1_g6-	Cd	828	F-box/kelch protein	*Camellia sinensis*
	DN44639_c0_g3 +	Cd	1244	Separase	*Actinidia chinensis*
	DN31795_c0_g1 +	Fe	651	Hypothetical protein TanjilG_03599	*Lupinus angustifolius*
	DN41780_c0_g1-	Cd	2666	Zinc finger protein-like	*Actinidia chinensis*
	DN38676_c0_g5-	Cd	1462	Transcription factor MYB26	*Vaccinium corymbosum*
	DN43227_c2_g4-	Cd	1237	LOB domain-containing protein	*Actinidia chinensis*
Stem	DN31674_c0_g1 +	Cd	2880	Glutaredoxin-like	*Actinidia chinensis*
	DN33390_c0_g2 +	Cd	1474	Reverse transcriptase zinc-binding domain-containing protein	*Artemisia annua*
	DN30630_c0_g3 +	Cd	990	PREDICTED: putative disease resistance RPP13-like protein 1 isoform X1	*Vitis vinifera*
	DN26228_c0_g4 +	Cd	1273	Hypothetical protein Ahy_B03g066511 isoform B	*Arachis hypogaea*
	DN22052_c0_g1-	Cd	652	Calcium/calmodulin-dependent/calcium-dependent protein kinase	*Trema orientale*
	DN45416_c0_g1 +	Cd	2040	L-ascorbate oxidase	*Actinidia chinensis*
	DN29755_c1_g4-	Cd	332	Hypothetical protein MIMGU_mgv1a009490mg	*Erythranthe guttata*
	DN23873_c1_g3-	Cd	1168	PREDICTED: NAC domain-containing protein 30 isoform X1	*Theobroma cacao*
	DN44665_c1_g1 +	Cd	4363	Galacturonosyltransferase	*Actinidia chinensis*
	DN36060_c0_g2-	Cd	1912	Non-characterized protein LOC107434963	*Ziziphus jujuba*
Leaf	DN41610_c1_g2 +	Cd	2467	UDP-glucose: flavonoid 3-O-glucosyltransferase	*Vaccinium corymbosum*
	DN33777_c2_g4 +	Cu	2497	RNA polymerase beta subunit	*Vaccinium macrocarpon*
	DN29488_c0_g1 +	Cu	2037	ABC transporter G family member 5-like	*Actinidia chinensis*
	DN35515_c0_g2 +	Cd	781	Phosphoenolpyruvate carboxylase	*Actinidia chinensis*
	DN23563_c0_g1 +	Cd	563	Glucan endo-1,3-beta-glucosidase	*Actinidia chinensis*
	DN25577_c0_g2 +	Cu	575	Low-temperature-induced protein	*Actinidia chinensis*
	DN23052_c0_g1 +	Cd	1302	Lon protease	*Actinidia chinensis*
	DN35869_c0_g2-	Cu	1327	Peroxidase	*Actinidia chinensis*
	DN24887_c0_g6-	Cu	1302	F-box/kelch-repeat protein	*Actinidia chinensis*
	DN41993_c0_g1-	Cu	1511	Glucan endo-1,3-beta-glucosidase	*Actinidia chinensis*
Fruit	DN41010_c1_g1 +	Zn	1855	UDP-rhamnose:rhamnosyltransferase	*Actinidia chinensis*
	DN43002_c0_g4 +	Mn	928	OleI	*Camellia oleifera*
	DN24275_c0_g4 +	Zn	539	Fatty acid desaturase	*Parasponia andersonii*
	DN43823_c0_g1 +	Mn	3530	Receptor-like protein kinase	*Actinidia chinensis*
	DN41309_c0_g1 +	Cu	1318	Late embryogenesis abundant protein D-34-like	*Cucurbita moschata*
	DN22211_c0_g1 +	Zn	971	BRASSINOSTEROID INSENSITIVE 1-associated receptor kinase	*Actinidia chinensis*
	DN41612_c1_g1-	Cd	2680	Protein DMP2-like	*Olea europaea*
	DN21404_c0_g1 +	Cu	1731	Predicted protein	*Hordeum vulgare*
	DN22677_c0_g1 +	Cu	1296	Heavy-metal-associated isoprenylated plant protein, partial	*Actinidia chinensis*
	DN21179_c0_g1 +	Mn	337	Ent-copalyl diphosphate synthase	*Actinidia chinensis*

+ is positive correlation,-is negative correlation, the same below

### Fungal diversity in the rhizosphere

#### Quality analysis of sequencing data

A total of 76 848 original data were obtained in the treatment group (M-Cd) and 78 259 original data were obtained in the control group (M-CK). After quality filtering, noise reduction, splicing and removal of chimeras, 64 661 sequences were obtained in M-Cd and 67 148 sequences were obtained in M-CK, accounting for 84.28 and 86.03% of the original sequences, respectively. For M-Cd and M-CK, 93.66 and 94.82% of the sequences, respectively, were 245–259 bp in length see Table 5 for detailed data.

**Table 5:** Sequencing results for rhizosphere fungi

Sample	Input	Filtered	Percentage of input that passed filter	Denoised	Merged	Percentage of input merged	Non-chimeric	Percentage of input non-chimeric	Length (245–259 bp)
M-Cd	76,848	65,999	86.05%	65,699	64,984	84.71%	64,661	84.28%	93.66%
M-CK	78,259	70,496	90.18%	70,198	69,345	88.71%	67,148	86.03%	94.82%

#### Taxonomic statistical analysis

According to the taxonomic statistics, M-Cd includes the annotated fungi, which are divided into 6 phyla, 15 classes, 31 orders, 55 families and 69 genera, with a total of 190 species. M-CK included 141 species of fungi, which were classified into 8 phyla, 14 classes, 34 orders, 44 families and 57 genera. M-Cd was stronger than M-CK with regard to family, genus and species classification, but weaker than M-CK with regard to phylum and order. The labeled M-Cd and M-CK samples included 67 common species at the level of species classification.

#### Relative species richness

**Fig. 6:**
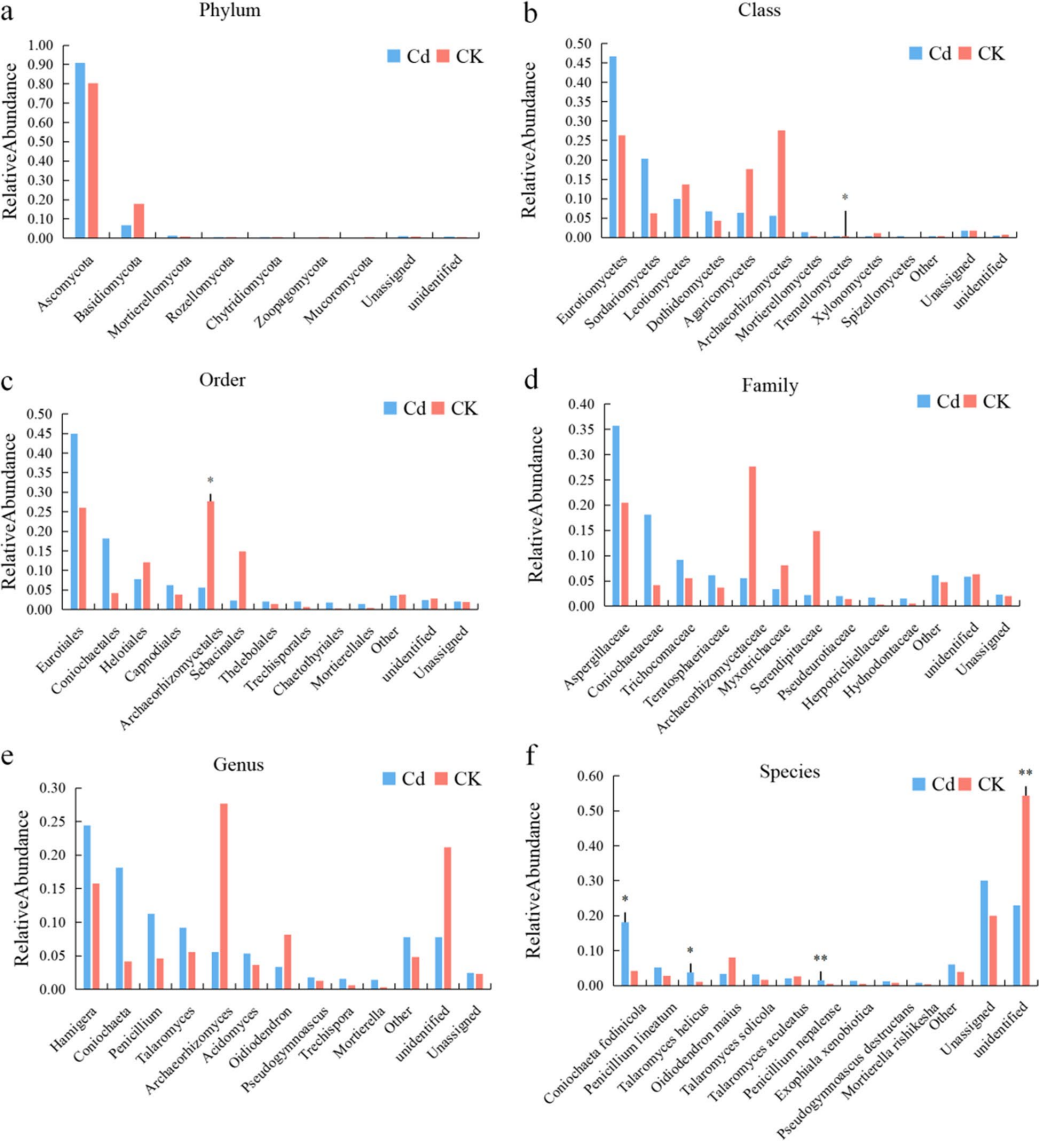
Relative abundance of blueberry rhizosphere fungi according to their natural classification system. * *P* < 0.05, * * *P* < 0.01

In order to simplify the presentation of the results, only the top 10 species of abundance level are shown, and the other species are combined into other, unclassified and unallocated species to represent the species that have not been annotated at this taxonomic level (Fig. 6) [40]. According to the classification method of Zhu et al., dominant microflora was defined as a relative abundance higher than 10%, and rare microflora was defined as a relative abundance of less than 0.01%. In order to better screen the differences in species between the two groups, *P* < 0.05 was used as the screening threshold of significant difference. At the phylum level, Ascomycota was the dominant microflora of M-Cd and M-CK, with richness index values of 0.9080 and 0.8039, respectively. Basidiomycota was also the dominant microflora of M-CK (Fig. 6a). At the class level, the dominant microflora of M-Cd and M-CK was Eurotiomycetes, the dominant microflora of M-Cd was Sordariomycetes, and the dominant microflora of M-CK included Leotiomycetes, Agaricomycetes and Archaeorhizomycetes. At the phylum level, although Tremellomycetes was not the dominant microflora of M-Cd and M-CK, the richness index of M-Cd was significantly different from that of M-CK (Fig. 6). At the order level, the dominant microflora of M-Cd consisted of Eurotiales and Coniochaetales, whereas the dominant microflora of M-CK included Eurotiales, Helotiales, Archaeorhizomycetes and Sebacinales, and the Archaeorhizomycetes richness index of M-CK was significantly higher than that of M-Cd (Fig. 6c). At the family level, the dominant microflora of M-Cd and M-CK was Aspergillaceae, and the dominant microflora of M-Cd was Coniochaetaceae (Fig. 6D). At the genus level, the dominant M-CK microflora only included *Hamigera*, but the dominant M-Cd microflora included *Hamigera*, *Coniochaeta* and *Penicillium* (Fig. 6e). At the species level, *Coniochaeta fodinicola* was the only dominant member of the microflora in M-Cd, but the richness index of *Talaromyces helicus* in M-Cd was significantly different from that in M-CK, and the richness index of *Penicillium nepalense* in M-Cd was significantly different from that in M-CK (Fig. 6F). It should be noted that there were significant differences in the richness of some species in M-Cd and M-CK at each taxonomic level, but they have not been included in Fig. 6 due to the low richness level.

#### Soil microbial diversity in the root zone

The alpha diversity indices at 97% similarity for M-Cd and M-CK are shown in Table 6. The Chao1 and ACE indices are used to measure species abundance (i.e. the number of species), and the Shannon and Simpson indices are used to measure species diversity [41]. It can be seen that the differences in the Chao1, ACE, Shannon and Simpson indices between M-Cd and M-CK are not significant, which indicates that there are no clear differences in soil microbial diversity between M-Cd and M-CK.

**Table 6:** Alpha diversity indices at 97% similarity for soil microbial diversity

Samples	Observed	Chao1	ACE	Shannon	Simpson	Coverage
M-Cd	171.33	171.33	171.41	3.13	0.12	1.00
M-CK	150.67	150.73	151.01	2.87	0.14	1.00
*P*-value	0.38	0.40	0.40	0.40	1.00	0.35

#### Correlation analysis between rhizosphere soil microbial richness and soil Cd levels

The results of the correlation analysis between soil Cd content and species richness are presented in Table 7; values of *R* ≥ 0.8 and *P* ≤ 0.05 were selected as the screening conditions. Two classes (Tremellomycetes and Chytridiomycetes), six orders (e.g. Filobasidiales), seven families (e.g. Conioscyphaceae), 12 genera (e.g. *Trichocladium*) and 6 species (e.g. *Trichocladium opacum*) were positively correlated with soil Cd level.

**Table 7:** Correlation between rhizosphere soil microbial classification and soil Cd levels

Class	Order	Family	Genus	Species
Tremellomycetes +	Filobasidiales +	Conioscyphaceae +	*Trichocladium*-	*Trichocladium opacum*–
Chytridiomycetes +	Conioscyphales +	Chytridiaceae +	*Conioscypha* +	*Coniochaeta fodinicola* +
	Chytridiales +	Rutstroemiaceae +	*Lambertella* +	*Lambertella tubulosa* +
	Eurotiales +	Vibrisseaceae +	*Cephalotheca* +	*Spirosphaera caricigraminis* +
	Archaeorhizomycetales-	Cephalothecaceae +	*Naganishia* +	*Cephalotheca sulfurea* +
	Chaetothyriales +	Filobasidiaceae +	*Chaetomium* +	*Talaromyces helicus* +
		Archaeorhizomycetaceae-	*Setophaeosphaeria*-	Unidentified–
			*Mrakia* +	
			*Ilyonectria* +	
			*Paecilomyces* +	
			*Archaeorhizomyces*-	
			*Talaromyces* +	

## Discussion

### Overall transcription of blueberry under Cd stress

Some studies have suggested that blueberry, a plant that grows in acid soil, is the dominant species in acid soil that is contaminated with heavy metals, but there has been little research on the mechanism of heavy metal tolerance in this species at the molecular level. The present study has utilized high-throughput sequencing technology, which can provide information about the response of plants to heavy metal stress. A total of 84 374 annotated genes, which included 3370 DEGs, were obtained from the root, stem, leaf and fruit tissue of blueberry plants that had been subjected to Cd stress. The DEGs were mainly involved in plant hormone signal transduction, ABC transport, the MAPK signal transduction pathway, starch and sucrose metabolism, riboflavin metabolism, porphyrin and chlorophyll metabolism, mismatch repair, photosynthesis, flavonoid biosynthesis and sphingolipid metabolism. The metabolic pathways of DEG enrichment also differed according to the specific tissue in which they were evaluated. For example, the DEGs in root tissue were mainly involved in glycosaminoglycan degradation, glycosphingolipid biosynthesis, sphingolipid metabolism, amino sugar and nucleotide sugar metabolism, and other degradation pathways related to cell wall synthesis and breakdown. The DEGs in stem tissue were associated with various defense responses (such as responses to water deficit, hydrogen peroxide and salt stress) and in signal transduction (such as the MAPK signal transduction pathway, plant hormone signal transduction and ABC transport). The DEGs in leaf tissue were mainly involved in photosynthesis, diterpenoid biosynthesis, circadian rhythms, oxidative phosphorylation, RNA polymerase activity, plant hormone signal transduction and flavonoid biosynthesis. The DEGs in fruit tissue were mainly associated with diterpenoid biosynthesis, oxidative phosphorylation, galactose metabolism, starch and sucrose metabolism, phenylpropanoid biosynthesis and carotenoid biosynthesis. A study of cadmium stress in cotton (*Gossypium hirsutum*) found that most DEGs were associated with catalytic activity and metal ion binding. Cotton responded to cadmium stress by increasing cell wall thickness, antioxidant activity and detoxification. It has been reported that the genes involved in preventing transport of Cd to rice grains include *OsIRT1*, *OsNramp*5, *OsVIT2*, *OsNRT1.5a* and *OsABCC1* [42]. Transcriptome analysis of the leaves and roots of *Broussonetia papyrifera* showed that Cd stress affected 47 KEGG pathways, mainly involving *bHLH*, *MYB* and other important transcription factors, plant–pathogen interactions, phenylpropanoid biosynthesis, plant hormone signal transduction, and other protein synthesis and metabolism processes [43]. In response to Cd stress, the main metabolic response of willow twig (*Panicum virgatum*) involves activation of redox-related metabolism, and 21 different shock transcription factors (HSFs) and 22 heat shock proteins (HSPs) are differentially expressed [44].These results are similar to the findings of the present study.

Most of the previous studies on the interaction between plants and heavy metals were conducted on the roots and leaves of herbaceous plants, and confirmed that the root system was the first organ to exhibit the effects of heavy metal toxicity, due to its direct exposure to heavy metals in the soil [22]. Interestingly, however, the results of the present study showed that in blueberry the DEGs were mainly concentrated in the stem, and the number of DEGs was lowest in root tissue (even lower than the number in leaf and fruit tissue). There were 13.7-fold more DEGs in stem tissue than in root tissue. The DEGs were involved in different metabolic pathways in each of these tissues, which indicated that there were also differences in the physiological responses of each type of tissue to Cd stress.

### Accumulation of and competition between heavy metals

Almost all plants accumulate essential heavy metals such as Fe, Mn, Cu and Zn in the body, and these heavy metals are also very important for the growth and development of plants. The absorption and accumulation of heavy metals by plants are dependent not only on the physical and chemical properties of the external environment, but also on the characteristics of the plant [45]. It has been shown that the levels of seven heavy metals in wheat grains are higher than those in maize grains. Moreover, as the heavy metal content of the soil increases, the biological concentration coefficient (BFC) of heavy metals in wheat and maize grains decreases exponentially [46]. When eggplant (*Solanum melongena*) was planted in polluted sludge, the heavy metal content of the roots was reported to be much higher than that of the buds and fruits, and the plant could more readily absorb Cd than either Pb or Ni from the soil [47]. These studies show that the type and content of heavy metals in different tissues vary due to differences in the transfer and fixation of heavy metals by plants.

In the study of heavy metal stress on blueberry, most of them are around the leaves and fruits [15, 48]. In a previous study, seedlings of the blueberry variety ‘Bluecrop’ accumulated Cd in the roots, but the Cd content of the stems was also very high, and the difference in Cd levels between the two tissues was not statistically significant [21]. In the present study, Cd was mainly accumulated in the stem, which was consistent with the finding of the highest number of DEGs in stem tissue, and also demonstrated the reliability of the sequencing results. The discrepancy in the results of the two studies may be due to the fact that the late-ripening blueberry variety ‘Bluegold’ was used in the present study, in order to prolong the stress treatment period, and different blueberry varieties accumulate Cd in different tissues. It can be confirmed that blueberry mainly accumulates Cd in the roots and stems, and the lowest level of accumulation is in the fruits, where the Cd concentration is much lower than that in the soil, roots, stems and leaves. The same research also showed that in *Vitis vinifera* the Cd content of the fruits of plants grown in polluted soil was lower than that of any other tissues [49]. However, the differences in heavy metal accumulation among different blueberry varieties need to be clarified in future research studies.

It has been widely reported that Cd^2+^ interacts with other metal cations in plants and soils [50]. In the present study we found that the Mn, Fe, Cu and Zn content of blueberry tissues was affected by Cd, and the diffusion of metal cations in soil was also affected by Cd^2+^. Mn was mainly absorbed by plants via active transport through the roots, and had an antagonistic effect on Cd accumulation [51]. External application of Mn could alleviate the toxicity of Cd to some extent [52], but the Mn content of blueberry roots was not affected by Cd, although the latter significantly reduced the transfer of Mn to stems, leaves and fruits. The passive diffusion of DGT showed that the Mn content increased with increasing Cd content, which indicated that although Cd had no effect on the Mn content of blueberry roots, the process of active absorption of Mn by roots may be affected.

Fe is an important element in chlorophyll synthesis, and an increase in Cd levels in leaves will lead to iron deficiency and chlorosis. An increase in Fe will inhibit the absorption of Cd [53], and sulfate (SO_4_^2–^) can promote the absorption of Fe by increasing competition [54]. However, in the present study, the increase in soil Cd concentration dramatically reduced the Fe content of blueberry roots, and to a lesser extent reduced that of leaves and fruits, but did not affect the Fe content of stems. The decrease in Fe content was due to the fact that Cd not only occupied the cation transport channel [55], but also inhibited the passive diffusion of Fe from the soil to the root tissue.

Cu has a role in the multi-channel oxidation process in plants, but there have been few studies on the interaction between Cu and Cd. It has been reported that the addition of excessive amounts of Cd to the culture medium can reduce the Cu and Ca content of the xylem in *Picea abies* [56]. Similar results have been obtained in studies of some grains and beans [57]. In the present study, too, severe Cd stress significantly reduced the Cu content of blueberry roots, stems and leaves, and even significantly decreased the passive absorption of Cu in soil.

Zn^2+^ in soil has been proved to be a competing ion for Cd^2+^ [58]. Zn has been reported to inhibit Cd accumulation in all organs of tomato (*Lycopersicon esculentum*) [59], but to significantly enhance Cd accumulation in petioles and reduce accumulation in the roots of *Potentilla griffithii* [60]. The Zn content of blueberry was strongly affected by plant exposure to Cd. Specifically, the Zn content of roots and leaves was significantly inhibited, but Zn accumulation in stems was significantly promoted, and the passive absorption of Zn in soil was also increased as Mn.

It is clear that treatment with Cd affected the Mn, Fe, Cu and Zn content of blueberry tissues, which is consistent with the findings of previous studies [24]. In blueberry under Cd stress, the Cd content of all tissues increased, although the magnitude of the increase varied widely. The Cd content was highest in stem tissue, followed by root and stem tissues (which had an almost identical Cd content), and was lowest in fruit tissue. As the Cd content of soil can be as high as 400 mg/kg, but the amount of Cd transferred from blueberry to fruit is very small (1 mg/kg), it is difficult to reach such a high level of Cd pollution in the soil in the external pollution area, so under the general pollution level, the content of Cd in fruit may be lower.

### Effects of heavy metals on rhizosphere microorganisms

Soil microorganisms are an important component of the soil ecosystem, especially for plants such as blueberry, which has roots with a low absorption capacity and uses fungal mycorrhiza to improve nutrient uptake and support growth. The interaction between soil microorganisms and soil environmental factors is extremely complex. In heavy-metal-contaminated soil, some microorganisms can modify, degrade or precipitate heavy metal compounds, while soil environmental factors can screen microbial communities [61]. In the present study, although the application of Cd did not have a significant impact on the biodiversity of soil fungi, it did significantly affect the dominant species of soil fungi. In fact, Cd is the preferred method of screening for soil fungi. At the phylum level, the two samples were mainly concentrated in the Ascomycota and Basidiomycota, while at the class level, growth of Agaricomycetes and Archaeorhizomycetes was inhibited by Cd, but growth of Eurotiomycetes and Sordariomycetes was promoted. At the order and family level, growth of both the Archaeorhizomycetales (of the Archaeorhizomycetes) and the Sebacinales (of the Agaricomycetes) was inhibited by Cd. The latter two orders and families have been demonstrated to be the dominant species in the mycorrhizal fungal community associated with blueberry [61], and fungi belonging to the order Sebacinales have the ability to replace ericoid mycorrhizae (ERM) associated with blueberry [29, 62]. In contrast, Cd stress promoted growth of fungi belonging to the Aspergillaceae and Trichocomaceae (of the Eurotiales) and the Coniochaetaceae (of the Coniochaetales). Some fungi belonging to these three families are highly tolerant of extreme environmental conditions, such as high temperature, high salt levels and high concentrations of heavy metals. For example, it has been reported that the dominant fungi in contaminated soil collected from an open pit mining area in Iglesiente (Sardinia, Italy) belong to the Trichocomaceae [63], while members of the Coniochaetaceae are heterotrophic ascomycetes that are widely distributed in terrestrial ecosystem, but are most numerous in extreme habitats [64]. Many species of the Coniochaetaceae are root endophytic fungi [65], but further studies are needed to determine whether they can be inoculated into blueberry roots to form endophytic fungi. At the taxonomic level of genus and species, some species with a high degree of adaptability to extreme environments have been preserved, such as *Coniochaeta fodinicola* and *Talaromyces helicus*, which are considered to be the dominant fungi in acid, industrial- and mining-contaminated soils, and are used to remove soil pollutants [66–68].

### Mechanism of Cd regulation in blueberry

**Fig. 7:**
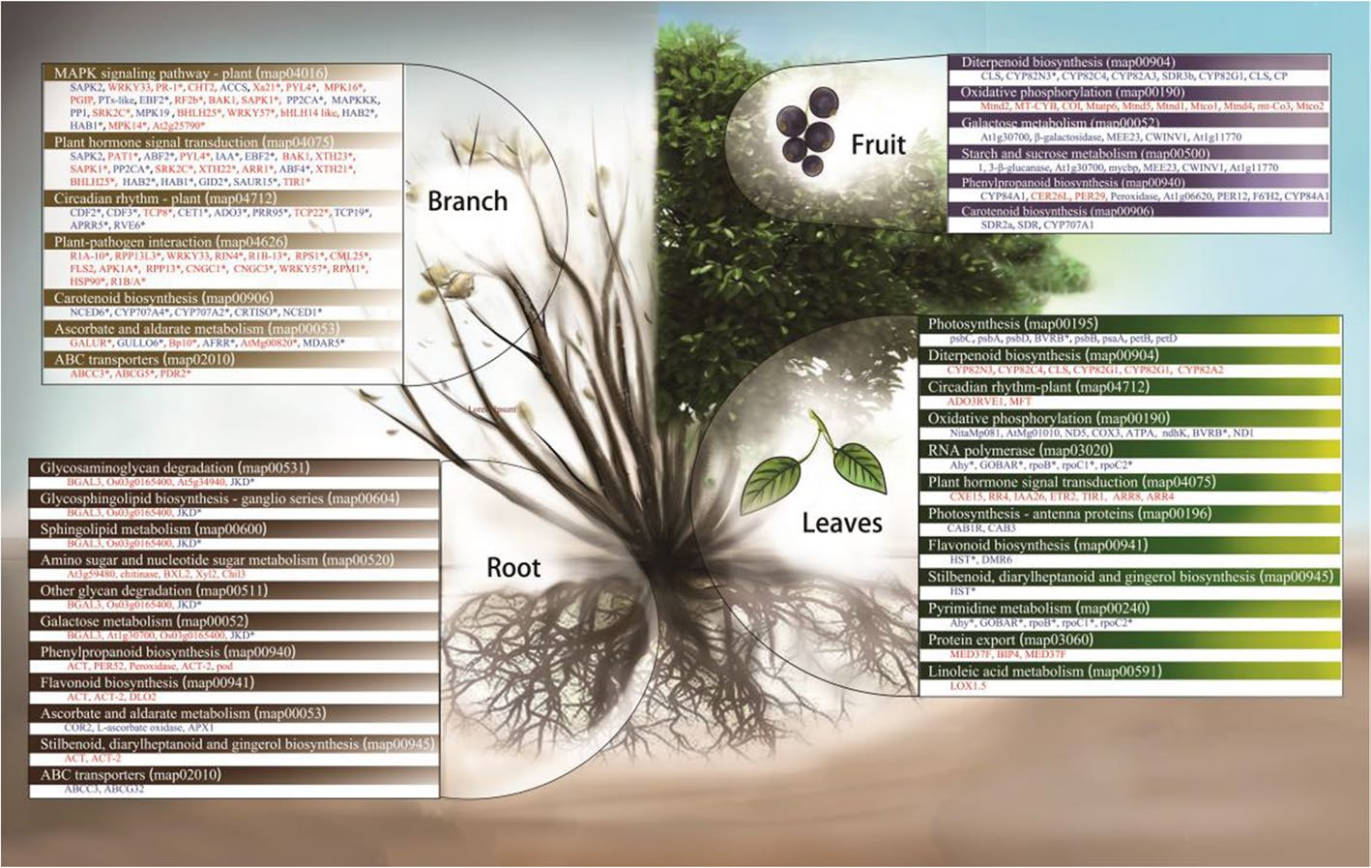
Differentially Expressed Genes in Differential Metabolic Pathways in Various Tissues of Blueberry. The red font is the up-regulated expression gene, and the blue font is the down-regulated expression gene. *It means that the difference is above the extremely significant level

After Cd entered the blueberry planting soil, it reflected the changes of the external environment of the root system. These changes altered the composition of the soil fungal community. The growth of several of the main mycorrhizal fungi of blueberry was inhibited, and some fungi that are resistant to extreme environmental conditions exhibited vigorous growth. At the same time, Cd altered the status of heavy metal absorption by the root system, which stimulated a series of reactions within the blueberry plant (Fig. 7).

First, Cd was absorbed along with other metal cations by the roots. When it had penetrated the root tissue by strong passive diffusion, reactions were induced in the root cell wall and cell membrane, such as sphingolipid metabolism (map00600), amino sugar and nucleoside sugar metabolism (map00520), other glycan degradation (map00511), and galactose metabolism (map00052). It was found that bgal3, os03g0165400, at5g34940, at3g59480, chitinase, bxl2, xyl2 and chil3 were up-regulated, but only JKD was down-regulated. All of these results indicated that the cell wall and cell membrane of blueberry root are important sites for Cd signal sensing, which is consistent with previous findings in studies on wheat(*Triticum aestivum*) [69]. The upward transport of Cd in blueberry resulted in its gradual accumulation in root and stem tissue. The stem tissue showed the strongest response to Cd, most markedly with the expression of *PR-1*, *Xa21*, *PYl4* and *MPK16* in the MAPK signaling pathway (map04016) and *ABF2*, *PYl4*, *IAA*, *EBF2*, *XTH23* and *SAPK1* in the hormone signaling pathway (map04075). At all levels of phosphorylation in the MAPK pathway the changes in MAPKK and MAPKKK led to the up-regulation of WRKY33 expression, which promotes an increase in *PAD3*, as has also been reported in *Arabidopsis thaliana* [70]. In addition, the response of blueberry to Cd stress is similar to its response to salt stress and drought stress. In the hormone signal transduction pathway, the up-regulated expression of abscisic acid receptor (PYL) leads to the up-regulated expression of serine/threonine protein kinase in response to Cd stress. The plant response to Cd stress also led to up-regulation of DEGs in the plant–pathogen interaction pathway (map04626). Cyclic nucleotide-gated channels (*CNGCs*) have an important role in control of the plant–pathogen interaction pathway [71]. The up-regulation of cngc1/3 in blueberry results in the expression of calcium-dependent protein kinase (*CDPK*), which affects the up-regulation of calmodulin (CaM/CML), *RPM1*, *RPS2*, *HSP90* and a series of disease-resistance-related genes. These genes jointly cause blueberry allergy. In addition, differential gene expression associated with phenylpropanoid biosynthesis (map00940), flavonoid biosynthesis (map00941), ascorbate and alginate metabolism (map00053) and carotenoid biosynthesis (map00906) also played a role in the Cd defense response of blueberry.

Several genes for ABC transporters (map02010) were up-regulated in blueberry stem, such as *ABCC3* and *ABCG5* [72]. ABCG-type ABC transporters were confirmed to be involved in plant hormone transport. When Cd was transported to blueberry leaves, the main effect was on the photosynthesis pathway (map00195), in which all DEGs were down-regulated (such as *PSBC*, *PSBA*, *PSBD* and *BVRB*), while all DEGs in the cytochrome P450 (*CYPs*) pathway of diterpenoid biosynthesis (map00904) were up-regulated. This indicated that the transfer of Cd to blueberry leaves had a marked effect on the photoperiod of the leaves, and the *CYPs* of blueberry leaves were involved in the regulation and detoxification of Cd. In the present study, the fruit was shown to be the tissue with the lowest Cd content. In the pathway of oxidative phosphorylation (map00190), *Mtnd2*, *MT-CY*B, *COI* and *Mtatp6* were up-regulated, while diterpenoid biosynthesis (map00904), oxidative phosphorylation (map00190), galactose metabolism (map00052), and starch and sucrose metabolism (map00500) were up-regulated. In the pathways of phenylpropanoid biosynthesis (map00940) and carotenoid biosynthesis (map00906), most of the DEGs were down-regulated. However, further research is needed to confirm the mechanism whereby fruit tissue inhibits the entry of Cd.

## Conclusions and future prospects

Blueberry, a perennial shrub that thrives in acid soil, is considered to be the dominant plant species in acid soils polluted with heavy metal compounds. However, the mechanisms of heavy metal absorption and metabolism in blueberry are unclear. Although high-throughput sequencing technology has enabled increasing numbers of studies on the mechanisms of plant regulation and metabolism of heavy metals, blueberry exhibits some unusual features. In response to Cd stress, the signal transduction, defense and detoxification mechanisms of this plant differ in each tissue type, reflecting the synergy and characteristics of each organization. In the present study, a total of 84 374 annotated genes were obtained from four tissue samples of blueberry by high-throughput sequencing technology, including 3370 DEGs, of which there were 184 DEGs in root tissue, 2521 DEGs in stem tissue, 360 DEGs in leaf tissue and 305 DEGs in fruit tissue. These DEGs were enriched in different metabolic pathways in different tissues. The Fe, Mn, Cu, Zn and Cd content of different tissues, soil and DGT of blueberry was determined by ICP-MS. It was found that the main Cd accumulation site was the stem, which was consistent with the sequencing results. The Cd content of stem tissue was higher than that of the other tissues, whereas the lowest Cd content was found in fruit tissue. Cd competed with other metal cations, and this severely affected the metabolism of other metal elements in blueberry tissues. Only through active absorption into blueberry roots, but also through a large number of passive diffusion into the roots. Cd in the soil also had a significant effect on the mycorrhizal fungi associated with blueberry. Growth of the dominant populations of mycorrhizal fungi, namely members of the Archaeorhizomycetes and Sebacinales, was inhibited, and members of the Coniochaetaceae, which can tolerate extreme environments, gradually became the dominant population. Thus, in the presence of the acid soil conditions that are necessary for growth of blueberry, Cd alters the growth environment of this plant and has toxic effects on its tissues. However, blueberry can resist the toxicity of Cd through its own metabolic regulatory mechanism.

The results of the present study, which demonstrate excessive accumulation of Cd and abundant DEGs in blueberry stem under Cd stress, point to interesting new avenues for future research. However, the most pressing need is for studies to improve our understanding of the long-distance transport of heavy metals in blueberry, the inhibition of Cd transport into fruit, the effect of Cd on the composition of the community of attached fungi and endophytic fungi in blueberry roots, and the regulation of Cd by bacteria in blueberry roots.

## Data Availability

The sequencing data has been submitted to NCBI Gene Expression Omnibus(GEO) database (https://www.ncbi.nlm.nih.gov/geo/) under the accession number of GSE168446 (https://www.ncbi.nlm.nih.gov/geo/query/acc.cgi?acc=GSE168446).

## Abbreviations

Cd: Cadmium
Zrt/IRT: Zinc iron regulatory protein
GSH: Glutathione
DEGs: Differentially expressed genes
qRT PCR: Quantitative real-time PCR
GAPDH: Glyceraldehyde-3-phosphate dehydrogenase
ICP-MS: Inductively coupled plasma mass spectrometry
DGT: Diffusive gradients in thin films
OTU: Operational taxonomic unit
GO: Gene ontology
KEGG: Kyoto Encyclopedia of Genes and Genomes
MAPK: Mitogen-activated protein kinase
HSFs: Shock transcription factors
HSPs: Heat shock proteins
BFC: Biological concentration coefficient
PYL: Abscisic acid receptor
*CNGCs*: Cyclic nucleotide-gated channels
*CDPK*: Calcium-dependent protein kinase
CaM/CML: Calmodulin
*CYP**s*: Cytochrome P450
GEO: Gene Expression Omnibus
